# Efficacy of Fibrin Glue in Skin Grafts for Skin Cancer (FiGSS): Open Randomised Clinical Trial

**DOI:** 10.1245/s10434-026-19428-0

**Published:** 2026-03-12

**Authors:** Ekta Paw, Rhys Jaa-Kwee, Mark Zonta, Venkat Vangaveti, Clare Heal

**Affiliations:** 1 College of Medicine and Dentistry, James Cook University, Douglas, Townsville, QLD Australia; 2 Department of Surgery, Townsville University Hospital, Douglas, QLD Australia

**Keywords:** Fibrin, Skin graft, Skin cancer, Randomised controlled trial, Split-skin graft

## Abstract

**Background:**

This study aimed to conduct the first randomized clinical trial on the use of fibrin glue in split-skin grafts for skin cancer. Fibrin glue is an accepted technique for affixing split-skin grafts. Evidence suggests that fibrin glue has greater effectiveness than sutures or staples, particularly in populations with more comorbidities.

**Methods:**

This randomized controlled clinical trial was conducted at a regional center in Queensland, Australia across the only two major hospitals in the area. The primary outcome assessed was graft take 1 month postoperatively. Pain with dressing changes and incidence of seroma, hematoma, and infection as well as operative time were assessed as secondary outcomes. Patients were recruited and randomized to either fibrin glue or staples and sutures for graft affixation. They were subsequently followed up at 1 week and 1 month for outcome assessment.

**Results:**

The study recruited 100 patients, and 83 patients with 133 grafts were analyzed for outcomes. Fibrin glue increased graft take by 17.13% when the study controlled for other variables. However, this was not statistically significant (*p* = 0.058; confidence interval [CI] − 0.63 to 34.89). There was a statistically significant reduction in the odds of seroma (odds ratio [OR] 0.08; CI 0.01–0.50; *p* < 0.01) and infection (OR 0.04; CI 0.00–0.33; *p* < 0.01) at 1 week.

**Conclusions:**

Fibrin glue may be of benefit for patients at higher risk for graft failure and is likely to benefit patients at increased risk of seroma and infection.

*Trial registration ANZCTR*: ACTRN12618000484246.

Fibrin glue has been used in many contexts intraoperatively. It was initially identified as a hemostatic agent that mimics the final events in the clotting cascade.^1–5^ When first developed, fibrin glue was prepared from patient plasma before use, but currently, commercial preparations with bovine protein are available with higher concentrations of fibrin.^6,7^

Fibrin glue has been investigated as an adhesive in various surgical disciplines.^8–10^ In split-skin-grafting, there is a suggestion that fibrin glue may improve graft take and other postoperative outcomes by adhering to the entire surface of the graft.^11–14^ Traditional fixation methods typically secure grafts at the margins with or without individual sutures in the central graft. This tends to leave areas of incomplete contact between the graft and wound bed. Comparatively, fibrin glue provides uniform adhesion, which improves the imbibition of nutrients from the graft bed before vascularization.^13,15^ This reduces the potential space for seroma formation and bacterial infection.^13,15^

Findings have shown that increased fibrin in wounds decreases the likelihood of graft failure and can induce angiogenesis.^16^ Therefore, fibrin glue may benefit split-skin-graft healing via several mechanisms. Very few reports describe adverse events after fibrin glue. Two reports of potential hypersensitivity and two reports of emphysema relating to incorrect application have been identified in the literature.^17–19^ Given its use across numerous surgical specialties, fibrin glue is likely both a safe and effective method to affix split-skin grafts.

Australia has high rates of skin cancer, with the state of Queensland as the skin cancer capital of the world.^20,21^ Wide oncologic resection of skin cancers requires reconstruction, of which split skin grafting is a commonly used technique.^22^

The current literature about skin grafts and fibrin glue focuses on its use in patients with burns, which is typically a younger population.^23,24^ Skin cancers are more common in the older adults, who have a higher incidence of comorbidities, which can affect wound healing.^20,25^ Fibrin glue has been investigated in other high-risk grafts, such as infected sites, mobile skin areas, and difficult-to-graft areas, with good results.^11,26–29^ Therefore, this technique may be of increased benefit for patients undergoing reconstruction with skin grafts after skin cancer resection. There is a paucity of prospective clinical trials examining the use of fibrin glue in skin grafts for skin cancer patients.^13,14^ To date, no clinical trials have examined the use of fibrin glue in skin grafts for this population.^14^

In addition to improving healing, fibrin glue may reduce operative time and pain in dressing changes for patients with split-skin grafts.^30^ Reduction in operative time may result from not needing to apply individual sutures or staples to a graft. Patients who require multiple dressing changes typically find it both inconvenient and painful, and an improvement in healing may reduce the number and duration of dressing changes.^31^ Therefore the use of fibrin glue has the potential for skin grafts to become quicker and less painful for patients.

The objective of this study was to examine the effectiveness of fibrin glue as an adhesive for split-skin grafts after skin cancer excision, which is of importance for any surgeon performing split-skin grafts for this population.

## Methods

Ethical approval for this study was granted by the Townsville Hospital Human Research Ethics Committee (approval HREC/17/QTHS/196). Representatives of the public and patients were present on the Ethics Committee reviewing the design and research. The full protocol for this trial has been published.^32^ The trial was registered with the Australian New Zealand Clinical Trials Registry (ACTRN12618000484246).

**Fig. 1:**
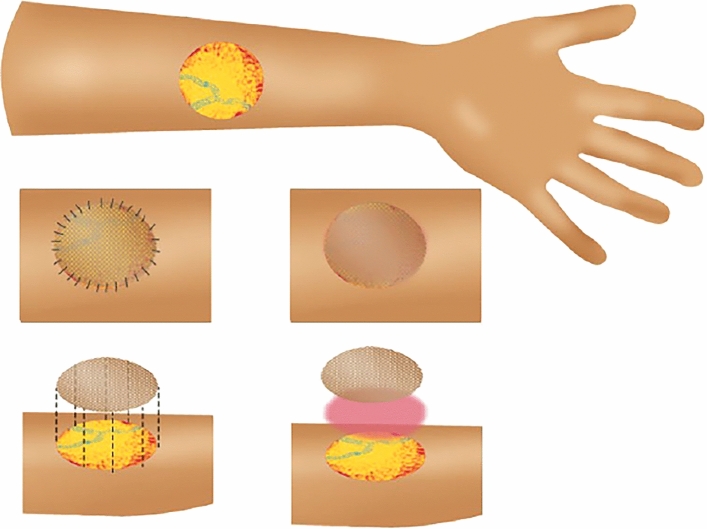
Affixation of skin graft with sutures (*left*)) and fibrin glue (*right*)

This study was conducted as a prospective randomized controlled superiority trial. The intervention group had fibrin glue applied to the graft bed intraoperatively as a thin even layer before graft-positioning. Dressings were applied with a standardized dressing protocol as pictorially represented in Fig. 1.^32^

The intervention used in this study was fibrin glue commercially available as two syringes containing human plasma-derived coagulation factors. The fibrin glue has had Australian Therapeutic Goods Administration approval since 2010.^33^ The treatment control group had grafts secured peripherally with sutures or staples and was recruited in parallel with the treatment group. The trial was conducted within the outpatient and surgical department of two sites (one public and one private hospital) in Townsville, Queensland, Australia.

All patients presenting to surgical clinics who required split-skin grafts were considered potential participants. The eligibility criteria specified patients who underwent surgery at one of the trial centers, had any histologic type of skin cancer, and were at least 18 years of age. Patients were excluded if they had an adverse reaction to fibrin glue in the past, had a hypersensitivity to the product, had skin grafts on digits or genitalia, or were pregnant.

The primary outcome measured was graft take 1 month after surgery as a percentage of each graft. This was assessed by the treating clinician and by an independent assessor via photographs. The secondary outcomes measured were operative time and pain at dressing changes (reported using Wong-Baker pain faces from 1 to 10 as integers), presence of infection, presence of hematoma, presence of seroma at 1 week and 4 weeks, and graft take at 1 week. Adverse effects were assessed intraoperatively at the time of fibrin glue administration and during follow-up appointments at 1 week and 1 month.

Sample size calculation was performed with a two-tailed analysis assuming a power of 0.80, an alpha of 0.05, and a clinically significant difference for the primary outcome of 15%.^32^ A sample size of approximately 300 was determined. Positive and negative stopping rules were in place for interim analyses.^32^

Randomization was performed at the patient level by the primary investigator with randomized blocks of two or four. Patient allocation was provided by sequential, opaque, sealed, tamper-proof envelopes by clinicians who could not access the full randomization sequence. Post-randomization blinding of clinicians and patients was impossible, so an independent outcome assessment was introduced.

### Statistical Analysis

Statistical analysis was completed in STATA 16.^34^ Demographic data were analyzed, and differences between treatment and intervention groups were analyzed using Fisher’s exact test for non-parametric data, chi-square for parametric data, and Mann-Whitney *U* for continuous non-parametric data. Multivariable regression analysis was used for outcome variables to control for differences between the treatment and intervention groups. Models were determined using forward selection and tests for covariance with variance inflation factor testing to determine collinear variables. Continuous outcome variables used linear regression with data transformations as appropriate. Binary or ordinal logistic regression was used for ordinal and binary variables such as pain (scored 1–10) and seroma. Goodness of fit and normality of residuals was checked for each model. Less than 10 % of data was missing for the primary outcome of interest and all secondary outcomes except pain at 1 month, for which 20 % of data was missing. Therefore imputation of missing values was not used. All secondary outcomes were pre-specified in the trial protocol.^32^

## Results

**Fig. 2:**
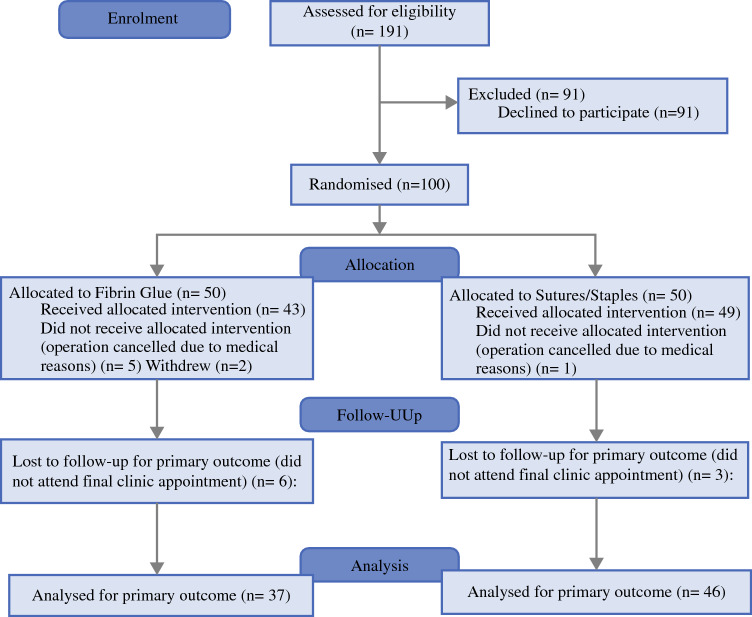
CONSORT 2025 flow diagram

Enrolment for the trial was open from June 2018 to November 2022, with final data collection in December 2022. During this time, 191 patients were assessed for eligibility, and 100 were randomized as specified in Fig. 2. Ultimately, 37 patients in the intervention arm and 46 patients in control arm were assessed for primary outcome. All patients analyzed received the intervention as per the randomization, so separate intention-to-treat and per protocol analyses were not required. Some of these patients had multiple grafts, and data were recorded for each graft site.

For the primary outcome measure, 133 grafts (70 in the intervention group and 63 in the control group) were analyzed. As per previous research with multiple wound sites in one patient, adjustment for clustering was not used because it was previously noted to have negligible effects on overall results.^35^ Analyzing per patient would have introduced aggregation issues. In discussion with the trial steering committee, recruitment was ceased early because of inability to achieve sufficient numbers. The COVID-19 pandemic led to significant restrictions on surgical services globally and in Queensland, and participant interest was lower than anticipated, potentially due increased hesitancy about participation in clinical research.

Intervention delivery was ensured by the operating surgeon and delivered as intended. One minor adverse event of slight graft-shearing immediately after surgery due to a patient accidentally removing dressings was reported. This was determined not to be related to the intervention.

### Demographic Data

Demographic data are summarized in Table 1, demonstrating no significant differences between the groups. Grafts all were ovoid in shape, so graft area was estimated by using documented width and length, and the area of an ellipse was calculated in cm^2^. Similarly, pack years were calculated using collected data on number of cigarettes smoked per day and years of smoking. Examples of other skin pathology include malignant eccrine tumor or atypical fibroxanthoma.

**Table 1:** Baseline data analysis

Demographic	Fibrin glue Intervention group (*n* = 37) *n* (%)	Sutures/staples Intervention group (*n* = 46) *n* (%)	*p* Value^a^
Age (years)	72.96 ± 11.56	74.56 ± 10.54	0.61
*Sex* Male Female	75 25	0.64 0.36	0.28
*Comorbidities* Hypertension Type 1 diabetes Type 2 diabetes Hypercholesterolemia Ischaemic heart disease Cerebrovascular disease Peripheral vascular disease	60 2 25 38 15 4 8	76 0 16 52 32 6 18	0.13 0.49 0.32 0.16 0.06 1.00 0.23
*Smoking status* Non-smoker Current smoker Ex-smoker Pack years	40 12 48 21.60 ± 16.66	52 14 34 32.04 ± 27.88	0.38 0.29

Graft characteristic	Fibrin glue Intervention group (*n* = 70) *n* (%)	Sutures/staples Intervention group (*n* = 63) *n* (%)	*p* value^a^
Graft area (cm^2^)	16.5 ± 12.8	19.8 ± 16.4	0.30
*Histopathology* SCC BCC Melanoma Other	51 31 12 5	50 24 24 1	0.17
*Graft site* Upper limb Lower limb Head Torso	7 68 23 2	22 59 19 0	0.08

SCC, squamous cell carcinoma; BCC, basal cell carcinoma

^a^Mann-Whitney *U* for continuous variables; Fisher’s exact test for categorical outcomes. Shapiro-Wilk and visual q-q plots used to test for normality

### Outcomes Analysis

Inter-rater reliability was assessed between clinically assessed graft takes by assessors who were not blinded and the independent outcome assessor who was blinded. Cohen’s kappa was 0.27 for week 4 graft take and 0.20 for week 1 graft take. Therefore, independent outcome assessments were used for the outcome analysis. Table 2 outlines direct comparisons between intervention groups without control for other confounders, which may have affected outcome. Table 3 outlines the results of multivariable regression analysis for the primary outcome of graft take at 4 weeks. All planned secondary outcome measures were analyzed except for hematoma and seroma at 1 month because there were fewer than five events limiting the ability to perform logistic regression. Fisher’s exact test was performed to determine whether there were any differences in additional treatment (oral antibiotics, drainage of seroma, or admission for intravenous antibiotics) between the two groups, and there were no significant differences at 1 week (*p* = 0.61) or 1 month (*p* = 0.21). Table 3 notes the results of multivariable analysis for secondary outcome measures and any variables with a statistically significant impact on the outcome of interest.

**Table 2:** All outcomes direct comparisons between intervention groups

Outcome (unit, *n*)	Fibrin glue (*n*)	Sutures/staples (*n*)	*p* Value^a^
Week 4 graft take (average %, 128)	75.10 ± 31.34 (67)	67.01 ± 33.52 (61)	0.03
Week 1 graft take (average %, 130)	82.94 ± 23.28 (68)	88.10 ± 11.24 (62)	0.63
Week 4 pain (average reported pain, 1–10, 109)	0.63 ± 1.53 (52)	1.70 ± 2.71 (57)	0.07
Week 1 pain (average reported pain 1–10, 116)	2.78 ± 2.77 (54)	3.24 ± 2.61 (62)	0.27
Week 4 hematoma (%, 131)	0 % (0)	0 % (0)	
Week 1 hematoma (%, 130)	0 % (0)	6 % (4)	0.07
Week 4 seroma (%, 131)	3 % (1)	2 % (2)	0.55
Week 1 seroma (%, 131)	10 % (6)	15 % (10)	0.27
Week 4 infection (%, 131)	10 % (6)	11 % (8)	0.45
Week 1 infection (%, 131)	17 % (11)	16 % (11)	0.51
Operative time (min, 129)	26.74 ± 19.18 (63)	28.79 ± 16.13 (66)	0.09

^a^Mann-Whitney *U* for continuous variables; Fisher’s exact test for categorical outcomes

**Table 3:** Multivariable regression analysis of primary outcome (percentage graft take at 4 weeks)

Variable	Coefficient (change to % graft take)	*p* value	Confidence interval
Fibrin glue (intervention)	17.13	0.058	–0.63 to 34.89
Hypertension	16.42	0.03	1.40 to 31.45
*Graft site* Upper limb Lower limb Head Torso	1 (Comparator) − 19.35 2.30 15.67	0.14 0.88 0.61	− 44.94 to 6.23 − 27.37 to 31.97 − 45.00 to 76.34
Cardiovascular disease	− 38.98	0.09	− 84.50 to 6.53
Smoking (pack years)	0.035	0.86	− 0.37 to 0.44
Type 2 diabetes mellitus	4.51	0.59	− 12.34 to 21.36
Ischemic heart disease	22.26	0.06	− 0.45 to 44.98

The intervention of fibrin glue was found to increase graft take by 17.13 % when the analysis controlled for other variables. However this result was not statistically significant (CI − 0.63 to 34.89; *p* = 0.058). At 1 week, the fibrin glue group did see a statistically significant reduction in the odds of seroma (OR 0.08; CI 0.01–0.50; *p* < 0.01) and infection (OR 0.04; CI 0.00–0.33; *p* < 0.01). Multivariable analysis for secondary outcomes is summarized in Table 4. Type 2 diabetes; smoking; presence of vascular disease, and graft size all were significant confounders of measured outcomes.

**Table 4:** Multivariable regression analysis of secondary outcomes

Outcome variables (*n*)	Coefficient/OR	***p*** Value	Confidence interval
Week 1 graft take (130) Fibrin glue (intervention) Age (cm^2^) Graft site–head Type 2 diabetes mellitus	Coeff − 1.56 − 0.45 13.49 − 18.44	0.61 < 0.01 0.01 < 0.01	− 7.56 to 4.44 − 0.75 to − 0.15 2.90 to 24.08 − 25.98 to − 10.90
Week 4 pain (109) Fibrin glue (intervention) Smoker Cerebrovascular disease Type 2 diabetes mellitus	OR 0.41 17.68 4.84 2.84	0.09 < 0.01 0.04 0.05	0.14–1.17 3.27–95.74 1.07–21.87 1.00–8.06
Week 1 pain (116) Fibrin glue (intervention) Type 2 diabetes mellitus Peripheral vascular disease	OR 0.53 3.54 0.34	0.11 0.01 0.05	0.24–1.15 1.30–9.63 0.12–0.98
Week 1 seroma (130) Fibrin glue (intervention) Hypertension	OR **0.08** 0.11	**<** **0.01** 0.02	**0.01**–**0.50** 0.02–0.72
Week 4 infection (131) Fibrin glue (intervention) Area (cm^2^)	OR 1.01 0.85	0.99 <0.01	0.17–6.07 0.75–0.96
Week 1 infection (131) Fibrin glue (intervention) Sex–female Type 2 diabetes mellitus	OR **0.04** 0.03 18.27	**< 0.01** < 0.01 < 0.01	**0.00**–**0.33** 0.00–0.26 2.85–117.03
Operative time (129) Fibrin glue (intervention) Area (cm^2^) Type 2 diabetes mellitus Smoking (pack years) Cerebrovascular disease Peripheral vascular disease	Coeff 0.86 1.01 1.80 0.99 1.73 1.46	0.27 0.05 < 0.01 < 0.01 0.03 0.04	0.66–1.13 1.00–1.02 1.42–2.27 0.98–1.00 1.05–2.85 1.02–2.07

Bold values indicate statistically significant change due to intervention.

OR odds ratio

## Discussion

The primary endpoint of graft take at 1 month did not reach a statistically significant threshold, but the data do suggest that after control for other comorbidities affecting graft take, there may be a clinically significant difference in graft take for patients in this population. Other studies examining fibrin glue have focused on burn populations, which generally are younger and have fewer comorbidities, and whereas fibrin glue has been noted as non-inferior, there may be a role for patients in this study population, for which the risk of graft failure is higher.^30,36–39^ The secondary outcome measures showed a reduction in the odds of seroma and infection at 1 week, which was statistically significant, and a clinically significant reduction in the odds. This again suggests a role for fibrin glue in patients at higher risk of seroma or infection, an idea supported by the current literature.^40^ Graft take at 1 week appeared to be reduced by application of fibrin glue, but this was neither a statistically nor a clinically significant change in the coefficient.

It was noted there were no significant demographic differences between the two intervention groups. The increased age of the study population compared with other studies on split-skin grafts is reflective of the population treated in regional Australia and the focus on skin cancer rather than burns (a commonly studied population in the split-skin grafting literature).^20,24,41,42^ The significant proportions of patients who had comorbidities also reflected the increased age of this study population. These comorbidities can significantly alter the postoperative course and must be accounted for in any wound-healing analysis.^43–45^

The results also indicate some other factors that significantly altered skin-grafting outcomes. The presence of hypertension appeared to increase graft take at 4 weeks, although this was when the analysis controlled for other vascular diseases, thus indicating patients for whom the sequelae of hypertension were not present. Therefore, this likely indicates an erroneous association. Grafts of the head had significantly increased take at 1 week, likely due to the increased collateral vasculature of the head and neck.^46^ Type 2 diabetes mellitus was shown to significantly decrease graft take at 1 week, significantly increase pain with dressing changes, and significantly increase the risk of infection. This is consistent with the current published literature on the microvascular complications of diabetes and their effects on wound-healing.^47^ Smoking also appeared to increase pain significantly with dressing changes at week 4.

Several statistically significant effects on operative time were observed. However, the interpretation of the coefficient was that an increase in each independent variable corresponded to less than a 2-min difference in operative time. Therefore, it is unlikely that any of these would be of any clinical significance.

### Study Limitations

This trial was limited by inability to recruit participants due to a decrease in ability to assess and treat surgical patients during the COVID-19 pandemic, an effect observed internationally.^48^ Lack of interest in participation also may have been affected by the spread of misinformation, reducing trust in medical institutions generally.^49^ Inability to reach the planned sample size for the population impacted the statistical significance of the primary outcome. It also was difficult to infer the effect of patients lost to follow-up evaluation on the outcomes. Due to the open nature of the trial, the primary outcome was assessed by independent assessment of photographs, but there may have been slight inaccuracy in assessing photographs versus clinically assessing the wound in person.

## Conclusions

Fibrin glue may offer some advantages over sutures and staples for split-skin-graft affixation in patients at higher risk of seroma and infection at their graft site. This includes older and more comorbid patients. There may be an additional benefit for patients who may not be able to travel long distances for suture removal. There may be an increase in overall graft take, but this study did not find the effect to be statistically significant. This is relevant to patients undergoing skin-grafting for skin cancer resection, or potentially any patients who are older and have more comorbidities, increasing the risk of graft failure.

### Future Directions

Clinical trials on the use of fibrin glue in skin-grafting still are sparse, particularly on the technique of reconstruction for skin cancer. More published data would be useful to pool for meta-analysis or a trial with increased power.

## Data Availability

De-identified participant data that underlie the results will be made available via James Cook University data repository.

## References

[1] Bergel S. Ueber Wirkungen des Fibrins. *Dtsch Med Wochenschr*. 1909;35:663–5.

[2] Spotnitz WD. Fibrin Sealant: past, present, and future: a brief review. *World J Surg*. 2010;34:632–4. 10.1007/s00268-009-0252-7. 19820991 10.1007/s00268-009-0252-7

[3] Currie L, Sharpe J, Martin R. The use of fibrin glue in skin grafts and tissue-engineered skin replacements: a review. *Plast Reconstr Surg*. 2001;108:1713–26. 11711954 10.1097/00006534-200111000-00045

[4] Brennan M. Fibrin glue. *Blood Rev*. 1991;5:240–4. 1782483 10.1016/0268-960x(91)90015-5

[5] Rousou J, Engelman R, Breyer R. Fibrin glue: an effective hemostatic agent for nonsuturable intraoperative bleeding. *Ann Thorac Surg*. 1984;38:409–10. 6333220 10.1016/s0003-4975(10)62297-7

[6] Kjaergard HK, Weis-Fogh US. Important factors influencing the strength of autologous fibrin glue; the fibrin concentration and reaction time: comparison of strength with commercial fibrin glue. *Eur Surg Res*. 1994;26:273–6. 7835384 10.1159/000129346

[7] Buckley R, Breazeale E, Edmond J, Brzezienski M. A simple preparation of autologous fibrin glue for skin-graft fixation. *Plast Reconstr Surg*. 1999;103:202–6. 9915185 10.1097/00006534-199901000-00033

[8] Hammond TM, Grahn MF, Lunniss PJ. Fibrin glue in the management of anal fistulae. *Colorect Dis*. 2004;6:308–19. 10.1111/j.1463-1318.2004.00676.x. 10.1111/j.1463-1318.2004.00676.x 15335361

[9] Koopman JE, Duraku LS, de Jong T, de Vries RBM, Michiel Zuidam J, Hundepool CA. A systematic review and meta-analysis on the use of fibrin glue in peripheral nerve repair: can we just glue it? *J Plast Reconstr Aesth Surg*. 2022;75:1018–33. 10.1016/j.bjps.2022.01.007. 10.1016/j.bjps.2022.01.007 35125308

[10] Panda A, Kumar S, Kumar A, Bansal R, Bhartiya S. Fibrin glue in ophthalmology. *Indian J Ophthalmol*. 2009;57. 10.4103/0301-4738.55079 PMC2804126 19700876

[11] Boeckx W, Vandevoort M, Blondeel P, Raemdonck D, Vandekerckhove E. Fibrin glue in the treatment of dorsal hand burns. *Burns*. 1992;18:395–400. 1445630 10.1016/0305-4179(92)90039-w

[12] McGill V, Kowal-Vern A, Lee M, et al. Use of fibrin sealant in thermal injury. Clinical Trial; Controlled Clinical Trial; Multicenter Study; Research Support; Non-U.S. Gov’t. *J Burn Carerehabil.* 1997;18:429–34. http://onlinelibrary.wiley.com/o/cochrane/clcentral/articles/915/CN-00143915/frame.html. 10.1097/00004630-199709000-00011 9313125

[13] Foster K, Greenhalgh D, Gamelli RL, et al. Efficacy and safety of a fibrin sealant for adherence of autologous skin grafts to burn wounds: results of a phase 3 clinical study. *J Burn Care Res*. 2008;29:293–303. 10.1097/BCR.0b013e31816673f8. 18354285 10.1097/BCR.0b013e31816673f8

[14] Paw E, Vangaveti V, Zonta M, Heal C, Gunnarsson R. Effectiveness of fibrin glue in skin graft survival: a systematic review and meta-analysis. *Ann Med Surg Lond*. 2020;56:48–55. 10.1016/j.amsu.2020.06.006. 32577231 10.1016/j.amsu.2020.06.006 PMC7305353

[15] Maeda M, Nakamura T, Fukui A, et al. The role of serum imbibition for skin grafts. *Plast Reconstr Surg*. 1999;104:2100–7. 10.1097/00006534-199912000-00023. 11149774 10.1097/00006534-199912000-00023

[16] Dvorak HF, Harvey S, Estrella P, Brown L, McDonagh J, Dvorak A. Fibrin containing gels induce angiogenesis. *Lab Invest*. 1987;57:673–86. 2447383

[17] Jankowski R, Beaudouin E, Kanny G, Wayoff M, Moneret-Vautrin DA. Hypersensitivity accidents to thrombin following the use of biological glues. *Annales d’oto-laryngologie et de chirurgie cervico faciale: bulletin de la Societe d’oto-laryngologie des hopitaux de Paris*. 1992;109(2):95–9. Accidents d’hypersensibilite a la thrombine, consecutifs a l’emploi de colles biologiques. 1524366

[18] Matsuse S, Maruyama A, Hara Y. Nitrogenous subcutaneous emphysema caused by spray application of fibrin glue during retroperitoneal laparoscopic surgery. *J Anesth*. 2011;25:426. 21424902 10.1007/s00540-011-1120-9

[19] Felema GG, Bryskin RB, Heger IM, Saswata R. Venous air embolism from Tisseel use during endoscopic cranial vault remodeling for craniosynostosis repair: a case report. *Pediatr Anesth*. 2013;23:754–6. 10.1111/pan.12180 23746210

[20] Cancer Council Australia. Skin cancer incidence and mortality. Cancer Council Australia. Retrieved 5 September 2025 at https://www.cancer.org.au/about-us/policy-and-advocacy/prevention/uv-radiation/related-resources/skin-cancer-incidence-and-mortality.

[21] Australian Institute of Health and Welfare. *Skin Cancer in Australia.* Vol. cat. no. CAN 96. . 2016. Australian Institute of Health and Welfare.

[22] Wilkinson D, Askew D, Dixon A. Skin cancer clinics in Australia: workload profile and performance indicators from an analysis of billing data. *Med J Aust*. 2006;184:162. 16489899 10.5694/j.1326-5377.2006.tb00176.x

[23] Shlash SO, Madani JO, Deib JI, et al. Demographic characteristics and outcome of burn patients requiring skin grafts: a tertiary hospital experience. *Int J Burns Trauma*. 2016;6:30–6. 27335695 PMC4913231

[24] Toppi J, Cleland H, Gabbe B. Severe burns in Australian and New Zealand adults: epidemiology and burn centre care. *Burns*. 2019;45:1456–61. 10.1016/j.burns.2019.04.006. 31053412 10.1016/j.burns.2019.04.006

[25] Australian Institute of Health and Welfare. 3.3 Chronic disease and comorbidities. Vol. Cat. no. AUS 199. 2016. Australia’s health 2016 Australia’s health series no 15

[26] Shetty S, Shetty M, Colaco A. Can use of fibrin glue in peridontal flap surgery be an alternative to suturing? *A review Int J Adv Res*. 2015;3:1573–5.

[27] Lilius P. Fibrin adhesive: its use in selected skin-grafting. *Scand J Plast Reconstr Surg*. 2009;21:245–8. 10.3109/02844318709086451. 10.3109/02844318709086451 2450397

[28] Vedung S, Hedlund A. Fibrin glue: its use for skin grafting of contaminated burn wounds in areas difficult to immobilize. *J Burn CareRehabil*. 1993;14:356–8. 10.1097/00004630-199305000-00009 8360243

[29] Jabs AD, Wider TM, DeBellis J, Hugo NE. The effect of fibrin glue on skin grafts in infected sites. *Plast Reconstr Surgy*. 1992;89:268–71. 10.1097/00006534-199202000-00011 1732894

[30] Paw E, Vangaveti V, Zonta M, Heal C, Gunnarsson R. Effectiveness of fibrin glue in skin graft survival: a systematic review and meta-analysis. *Ann Med Surg*. 2020;56:48–55. 10.1016/j.amsu.2020.06.006. 10.1016/j.amsu.2020.06.006 PMC7305353 32577231

[31] Sinha S, Schreiner AJ, Biernaskie J, Nickerson D, Gabriel VA. Treating pain on skin graft donor sites: review and clinical recommendations. *J Trauma Acute Care Surg*. 2017;83. 10.1097/TA.0000000000001615 28598907

[32] Paw E, Vangaveti V, Zonta M, Heal C. Protocol for an open randomised controlled trial investigating Fibrin Glue in Skin grafts for Skin cancer (FiGSS). *BMJ Open*. 2022;12:e064431. 10.1136/bmjopen-2022-064431. 36351733 10.1136/bmjopen-2022-064431 PMC9644346

[33] Therapeutic Good Administration. ARTISS fibrin sealant VH S/D 4 IU (frozen) solution for sealant syringe (163515). Retrieved 29 September 2025 at https://www.tga.gov.au/resources/artg/163515.

[34] Stata Statistical Software: Release 16. StataCorp LP; 2019.

[35] Heal CF, Banks JL, Lepper PD, Kontopantelis E, van Driel ML. Topical antibiotics for preventing surgical site infection in wounds healing by primary intention. *Cochrane Database Syst Rev*. 2016. 10.1002/14651858.CD011426.pub2. 27819748 10.1002/14651858.CD011426.pub2 PMC6465080

[36] Boccara D, De Runz A, Bekara F, Chaouat M, Mimoun M. Artiss Sealant®: an alternative to stapling skin grafts on the dorsal side of the hand and fingers. *J Burn Care Res*. 2017;38:283–9. 10.1097/BCR.0000000000000503. 28181985 10.1097/BCR.0000000000000503

[37] Boeckx W, Vandevoort M, Blondeel P, Van Raemdonck D, Vandekerckhove E. Fibrin glue in the treatment of dorsal hand burns. *Burns*. 1992;18:395–400. 10.1016/0305-4179(92)90039-W. 1445630 10.1016/0305-4179(92)90039-w

[38] Dahlström KK, Weis-Fogh US, Medgyesi S, Rostgaard J, Sörensen H. The use of autologous fibrin adhesive in skin transplantation. *Plast Reconstr Surg*. 1992;89:968–72. 10.1097/00006534-199205000-00034. 1561270

[39] Greenhalgh DG, Gamelli RL, Lee M, et al. Multicenter trial to evaluate the safety and potential efficacy of pooled human fibrin sealant for the treatment of burn wounds. Clinical Trial; Multicenter Study; Randomized Controlled Trial; Research Support, Non-U.S. Gov’t. *J Trauma*. 1999;46:433–40. http://onlinelibrary.wiley.com/o/cochrane/clcentral/articles/973/CN-00160973/frame.html. 10.1097/00005373-199903000-00014 10088846

[40] Seretis K, Bounas N. Securing skin grafts: a network meta-analysis. *J Plast Reconstr Aesth Surg*. 2024;96:146–57. 10.1016/j.bjps.2024.07.005. 10.1016/j.bjps.2024.07.005 39089211

[41] Gayova M, Babik J, Lengyel P, Katuchova J, Gazda J. Baseline factors associated with split-thickness skin graft failure in burn patients: a retrospective observational analysis of a cohort of 69 burn patients. *Eur J Plast Surg*. 2023;46:619–24. 10.1007/s00238-023-02049-1.

[42] Tracy LM, Singer Y, Schrale R, et al. Epidemiology of burn injury in older adults: an Australian and New Zealand perspective. *Scars Burns Healing*. 2020;6:2059513120952336. 10.1177/2059513120952336. 33062309 10.1177/2059513120952336 PMC7534068

[43] Dryden M, Baguneid M, Eckmann C, et al. Pathophysiology and burden of infection in patients with diabetes mellitus and peripheral vascular disease: focus on skin and soft-tissue infections. *Clin Microbiol Infect*. 2015;21:S27-32. 10.1016/j.cmi.2015.03.024. 26198368 10.1016/j.cmi.2015.03.024

[44] Phillips P, Sampson E, Yang Q, Antonelli P, Progulske-Fox A, Schultz G. Bacterial biofilms in wounds: chronic wounds. *Wound Healing Southern Africa*. 2008;1:10–2. 10.10520/EJC82729.

[45] Ramanujam CL, Han D, Fowler S, Kilpadi K, Zgonis T. Impact of diabetes and comorbidities on split-thickness skin grafts for foot wounds. *J Am Podiatr Med Assoc*. 2013;103:223–32. 10.7547/1030223. 23697729 10.7547/1030223

[46] Turowski B, Zanella FE. Interventional neuroradiology of the head and neck. *Neuroimaging Clin*. 2003;13:619–45. 10.1016/S1052-5149(03)00047-9. 10.1016/s1052-5149(03)00047-9 14631695

[47] Greenhalgh DG. Wound healing and diabetes mellitus. *Clin Plast Surg*. 2003;30:37–45. 10.1016/S0094-1298(02)00066-4. 12636214 10.1016/s0094-1298(02)00066-4

[48] Søreide K, Hallet J, Matthews JB, et al. Immediate and long-term impact of the COVID-19 pandemic on delivery of surgical services. *Br J Surg*. 2020;107:1250–61. 10.1002/bjs.11670. 32350857 10.1002/bjs.11670 PMC7267363

[49] Roozenbeek J, Schneider CR, Dryhurst S, et al. Susceptibility to misinformation about COVID-19 around the world. *Royal Soc Open Sci*. 2020;7:201199. 10.1098/rsos.201199. 10.1098/rsos.201199 PMC7657933 33204475

